# Bone marrow mesenchymal stem cells tune the differentiation of myeloid-derived suppressor cells in bleomycin-induced lung injury

**DOI:** 10.1186/s13287-018-0983-1

**Published:** 2018-09-26

**Authors:** XiaoSan Su, Liu Yang, YanFeng Yin, Jie Huang, Fei Qiao, Yu Fang, Lu Yu, YinYin Wang, KaiHua Zhou, Jun Wang

**Affiliations:** 1 Biomedical Research Center, Affiliated Calmette Hospital of Kunming Medical University, 504 Qing Nian Road, Kunming, Yunnan 650011 People’s Republic of China; 2 Department of Anesthesiology, First Affiliated Hospital of Kunming Medical University, 295 Xi Chang Road, Kunming, Yunnan 650032 People’s Republic of China; 3 Department of Pathology, Affiliated Calmette Hospital of Kunming Medical University, 504 Qing Nian Road, Kunming, Yunnan 650011 People’s Republic of China; 4 Department of Respiratory Diseases, Affiliated Calmette Hospital of Kunming Medical University, 504 Qing Nian Road, Kunming, Yunnan 650011 People’s Republic of China

**Keywords:** Bone marrow mesenchymal stem cells, Myeloid-derived suppressor cells, Bleomycin, Fibrosis

## Abstract

**Background:**

Bone marrow mesenchymal stem cells (BMSC) transfer has been attempted as a therapeutic strategy in experimental lung injury and fibrosis. Reduction of neutrophilic infiltration is one of the mechanisms involved in this effect. However, the mechanisms by which BMSC modulate neutrophil remains unknown.

**Methods and results:**

Exposure of mice to bleomycin (BLM) resulted in significant accumulation of cells that express neutrophilic markers Gr-1^High^CD11b^+^Ly-6G^High^F4/80^―^CD115^―^CD49d^―^. These cells lacked immunosuppressive activity and could not be defined as myeloid-derived suppressor cells (MDSC). When BMSC were administrated to BLM-treated mice, they tuned the differentiation of Gr-1^High^CD11b^+^ toward Gr-1^Low^CD11b^+^ cells. Gr-1^Low^CD11b^+^ cells exhibited unsegmented nuclei and expressed F4/80, Ly-6C, CD49d, and CD115 markers. These cells had potent immunosuppressive activity and thus could be defined as monocytic MDSC. As a result of such immunoregulation, BMSC mediated a decrease of pro-inflammatory products and amelioration of lung injury in BLM-treated mice. Further study using antibody array showed increased expression of macrophage colony-stimulating factor (M-CSF) in BMSC-treated mice. Accumulation of Gr-1^Low^CD11b^+^ cells in BMSC-treated mice was abrogated in M-CSF neutralizing mice. The beneficial effect of BMSC was independent of the ability of the cells to engraft in lung and in vitro coculture study of BMSC with Gr-1^+^CD11b^+^ cells showed that the induction of Gr-1^Low^CD11b^+^ cells by BMSC was independent of cell-cell contact.

**Conclusions:**

These results document the generation of Gr-1^High^CD11b^+^ cells in BLM-treated mice, and suggest that BMSC tune the differentiation of Gr-1^High^CD11b^+^ toward Gr-1^Low^CD11b^+^ cells and therefore inhibit the progression of BLM-induced lung injury.

**Electronic supplementary material:**

The online version of this article (10.1186/s13287-018-0983-1) contains supplementary material, which is available to authorized users.

## Background

Pulmonary fibrosis is characterized by epithelial cell injury and hyperplasia, variable degrees of inflammatory cell infiltrate, fibroblast proliferation and accumulation, and deposition of extracellular matrix [1]. The end results of this process are a loss of alveolar surface area and lung elasticity, leading to severe compromises in pulmonary function and respiratory failure [2]. One of the mechanisms that have been implicated in lung injury and fibrosis progression is neutrophil infiltration. In human, high numbers of neutrophils were found in bronchoalveolar lavage (BAL) fluids of patients with severe pulmonary fibrosis disease [3–5]. In mice, bleomycin (BLM) induces lung epithelial cell death, followed by acute neutrophilic influx, chronic inflammation, and parenchymal fibrosis [6]. An association between accumulation of neutrophils and development of injury and fibrosis in mice induced by BLM has been demonstrated [7–9], further supporting the impact of these cells in lung injury and fibrosis.

Myeloid-derived suppressor cells (MDSC) are characterized by their myeloid origin, immature state, and most importantly by their potent ability to suppress immune responses, especially T cell proliferation and cytokine production [10]. MDSC consist of two large groups of cells: granulocytic MDSC (G-MDSC) and monocytic MDSC (M-MDSC) [11, 12]. G-MDSC are phenotypically and morphologically similar to neutrophils, whereas M-MDSC are similar to monocytes. These cells represent a pathologic state of activation of monocytes and relatively immature neutrophils. MDSC are rare in steady-state conditions, but they accumulate abundantly during different pathologies and contribute to their progression. Recent studies highlight an emerging role for MDSC in pulmonary diseases. It has been shown that G-MDSC accumulate in cystic fibrosis (CF) patients, particularly in patients infected with Pseudomonas aeruginosa and correlate with CF lung disease activity [13]. While the role of MDSC in cancer has been studied in depth, our understanding of their relevance for pulmonary injury/fibrosis has just begun to evolve.

During the past few years, bone marrow mesenchymal stem cells (BMSC) transfer has been attempted as a therapeutic strategy in experimental lung injury and fibrosis. One alternative mechanism is that BMSC may change the microenvironment of the lungs, possibly by modulating the production of soluble factors, including transforming growth factor-β (TGF-β), interleukin (IL)-1, and IL-6, all of which are considered to be possible mediators of lung injury and fibrosis [14–16]. A systematic review demonstrates that the total number of cells in BAL was assessed in five studies of mesenchymal stem cell (MSC) therapy in animal models of BLM-induced pulmonary injury/fibrosis, four of which found it to be decreased by MSC therapy [17–21]. Meanwhile, neutrophil count in BAL was assessed in four studies, all of which found it to be decreased by MSC therapy [17–20]. However, few studies have demonstrated the mechanisms involved in modulatory effects of MSC on BLM-induced neutrophil recruitment and inflammatory cytokine expression.

Endotracheal challenge in mice with BLM represents a well-established animal model of acute lung injury resulting in pulmonary fibrosis. Using this experimental model, we tested the hypothesis that MDSC are directly involved in the development of experimental lung injury. We also evaluated the modulatory effects of BMSC transfer on the development of MDSC in BLM-induced lung injury.

## Methods

### BMSC culture

C57BL/6 mice were sacrificed by heart injection of KCL and then femora and tibiae were isolated. After dissection of attached muscle and connective tissue from the bones, the marrow was extruded by clipping of the epiphyseal ends of the bones and flushing using a needle with α-MEM (GibcoBRL, Gaithersburg, MD, USA), supplemented with 10% heat-inactivated fetal calf serum (FCS), β-mercaptoethanol (5 × 10^− 5^ mol/L), penicillin (100 U/mL) and streptomycin (100 μg/mL) (BMSC media). The marrow was plated in tissue culture flasks, and nonadherent hematopoietic cells were removed at day 3, followed by BMSC media replenishment every 3 days. Adherent BMSC were harvested and passed at low density (100–200 cells/cm^2^) and maintained in a humidified incubator (37 °C; 5% CO_2_) under subconfluent conditions to prevent cell differentiation. The phenotypes of BMSC were examined using the following rat anti-mouse monoclonal antibodies (mAb): FITC-conjugated CD34 (Clone RAM34) and CD45 (Clone 30-F11), PE-conjugated CD44 (Clone IM7) and CD90 (Clone OX-7) (BD Pharmingen, San Diego, CA, USA). The phenotypes of BMSC were shown to be for CD34^-^, CD45^-^, CD44^+^, and CD90^+^ (Additional file 1: Figure S1a). Potentials of mouse BMSC were evaluated as differentiation into adipocytes and osteocytes (Additional file 1: Figure S1b). Human BMSC were purchased from Cyagen Biosciences (Suzhou, China) and cultured according to the manufacturer’s instructions.

### Induction of lung injury with BLM and treatment with BMSC

Specific pathogen-free, 8-week-old female C57BL/6 mice were randomly divided into three groups: sham, BLM, and BMSC. Mice in the sham group were injected intratracheally (i.t.) with 30 μL normal saline; the others were injected i.t. with 5 mg/kg BLM in 30 μL normal saline. Twenty-four hours after BLM treatment, 0.1 mL normal saline (BLM group) or BMSC suspension (1 × 10^7^ cells/mL) (BMSC group) was injected through the tail vein. The day of intratracheal injection with BLM or saline was designated as day 0. On days 0, 3, 7, 14, 21, and 28, six mice from each group were sacrificed. All animal experiments were performed according to the guidelines and protocols approved by the Institutional Animal Care and Use Committee at Kunming Medical University (Kunming, China).

### Bronchoalveolar lavage (BAL) and preparation of the lung tissues for analysis of the dry/wet ratio and histologic examination

After the mice were killed, the left main bronchus was tied with a string and the right lung was removed for cytokines and histologic analysis. The left lung lobes were removed for wet/dry ratio analysis as previously described [22]. After the wet weight of the excised left lobe was measured, the lobe was placed with a desiccant in an oven at 60 °C and reweighed 4 days later. BAL was performed using 1 mL infusion of phosphate-buffered saline (PBS) into right lung with withdrawal via a cannula inserted into the trachea. The BAL fluid was centrifuged at 500 g for 10 min and the supernatant was stored at −70 °C. The right lung was flushed with PBS and fixed in 4% paraformaldehyde for at least 24 h and paraffin embedded. Paraffin-embedded lungs were serially sectioned and histologically examined with hematoxylin and eosin (H&E) stain.

### Measurement of collagen using Masson’s trichrome stain and Sircol collagen assay

Mouse lung tissues were processed for paraffin embedding, and serial sections were stained with Masson’s trichrome as previously described [23]. The total amount of soluble collagen was measured using a Sircol collagen assay kit according to the manufacturer’s instructions (Biocolor, Carrickfergus, Northern Ireland, UK).

### Flow cytometry (FCM) analysis and cell sorting

Suspensions of lung cells were prepared using an enzyme digestion method [24]. Lungs were perfused with 0.02% EDTA-PBS to wash blood vessels and incubated in RPMI 1640 medium containing collagenase/DNase I, and cell suspensions were washed. Cells were stained with different combinations of the following rat anti-mouse mAbs: FITC-CD11b (clone M1/70.15), PE-Gr-1 (clone RB6-8C5), PE-Cy5-Ly-6G (clone 1A8), PE-Cy5-Ly-6C (clone HK1.4), PE-Cy5-F4/80 (clone BM8), PE-Cy7-CD49d (clone R1–2), and PE-Cy7-CD115 (clone 604B5 2E11) (BD Pharmingen). For cell sorting, suspensions of lung cells were stained with FITC-CD11b and PE-Gr-1. Isolation of Gr-1^+^CD11b^+^, Gr-1^High^CD11b^+^ and Gr-1^Low^CD11b^+^ cells was performed on FACSVantage SE cell sorter (Becton Dickinson, Franklin Lakes, NJ, USA).

### Measurement of the IL-1β, IL-6, TGF-β, TNF-α and VEGF

The interleukin-1β (IL-1β) (Invitrogen, Waltham, MA, USA), IL-6, transforming growth factor beta (TGF-β), tumor necrosis factor alpha (TNF-α) and vascular endothelial growth factor (VEGF) (R&D Systems, Minneapolis, MN, USA) levels in BAL fluid were measured using enzyme-linked immunosorbent assay (ELISA) kits according to the manufacturers’ protocols. The minimum detection limits for IL-1β, IL-6, TGF-β, TNF-α, and VEGF were 3.9 pg/ml, 7.8 pg/ml, 31.25 pg/ml, 0.78 pg/ml, and 7.8 pg/ml, respectively.

After BMSC treatment in BLM-treated mice, Gr-1^+^CD11b^+^, Gr-1^High^CD11b^+^ or Gr-1^Low^CD11b^+^ cells in the lungs of sham-, BLM- or BMSC-treated mice were isolated by fluorescence-activated cell sorting (FACS) and cultured in 24-well plate (1 × 10^5^ cell per well). Twenty-four hours later, supernatants were collected and the levels of IL-1β, IL-6, TGF-β, TNF-α, and VEGF were measured using ELISA.

### MDSC suppression assay

The suppressive function of MDSC was assessed based on their ability to inhibit CD3 engagement-induced T cell proliferation. CD3^+^ cells were isolated from spleens of naïve C57BL/6 mice using anti-CD3 magnetic beads (Miltenyi Biotec, Bergisch Gladbach, Germany) and plated at 2 × 10^5^ cells/well in 1 μg/mL of rat anti-mouse CD3 mAb (BD Pharmingen)-coated plates. Isolated Gr-1^+^CD11b^+^, Gr-1^High^CD11b^+^ or Gr-1^Low^CD11b^+^ cells (1 × 10^5^ cells/well) from sham-, BLM- or BMSC-treated mice were added to the wells. Cell proliferation was determined 72 h later after incubating with ^3^H-thymidine for the last 16 h.

### Immunoassay for cytokines

The relative expression levels of 40 mouse cytokines were determined in serum using a Mouse Cytokine Array Panel A (Catalog Number ARY006, Lot 331,103) (R&D Systems). Mixed serum was collected from the BLM or BMSC group. Pixel densities on developed X-ray film could be collected and analyzed using a transmission-mode scanner (Bio-Rad, Hercules, CA, USA) and Image-Lab Software (Bio-Rad). The concentration of macrophage colony-stimulating factor (M-CSF) in serum was detected with an ELISA kit (RayBiotech, Norcross, GA, USA) according to the manufacturer’s instructions.

### Neutralization of M-CSF in vivo

The monoclonal anti-M-CSF antibody (clone 131,621) (Thermo Fisher Scientific, Rockford, IL, USA) was purchased and provided by Prof. S. Zhang (Department of Immunology, Cancer Institute, Peking Union Medical College and Chinese Academy of Medical Sciences, Beijing, China). C57BL/6 mice were treated with 5 mg/kg BLM via intratracheal instillation. BMSC (1 × 10^6^ cells/mouse) were administered via tail vein 24 h after BLM treatment. For neutralization of M-CSF, BMSC-treated mice were injected introperitoneally (i.p.) with 10 μg of rat anti-mouse M-CSF mAb or an isotype mAb on days 0, 2, 4, and 6 after BMSC infusion. On days 0, 3, 7, 14, 21, and 28, six mice from each group were sacrificed. Lung specimens were collected and the proportions of Gr-1^High^CD11b^+^ and Gr-1^Low^CD11b^+^ cells in lungs were analyzed by FCM.

### PCR analysis for detection of allogeneic BMSC

Female C57BL/6 mice were injected i.t. with 5 mg/kg BLM in 30 μL normal saline. Twenty-four hours after BLM treatment, male BMSC (1 × 10^6^ cells/mouse) were injected through the tail vein. Peripheral blood and lung samples were collected at 24, 48, 72, 96, and 120 h after BMSC administration. Total DNA was extracted from lung tissue and peripheral blood using a Bio Robot EZ1 (Qiagen, Hilden, Germany) and the EZ1 Tissue Kit (Qiagen) according to the manufacturer’s instructions, and was then amplified in 50 μL reactions containing dNTPs (200 μmol) and GoTaq DNA polymerase reagents (Promega, Madison, WI, USA) and the following primers (25 pmol) specific for the murine sequence of the sex determination region of the Y chromosome (*SRY*) gene: 5’-GTCAAGCGCCCCATGAATGCAT-3′ (forward) and 5’-AGTTTGGGTATTTCTCTCTGTG-3′ (reverse). PCR products were analyzed by agarose gel electrophoresis and ethidium bromide staining.

The transcript levels of the *SRY* gene were determined using a quantitative reverse transcript PCR (RT-qPCR). Briefly, total RNA was isolated from lungs and peripheral blood of BMSC-treated mice using the RNA Easy Mini Kit (Qiagen, Valencia, CA, USA), and then reverse transcribed at 42 °C for 1 h in a 50 μL reaction mixture using the Moloney-Murine Leukemia Virus Reverse Transcriptase (M-MLV-RT, Promega, Madison, WI, USA) and oligo-dT15 primer. Sequences of the primers used for RT-PCR amplification: 5’-AGCTCTTACACTTTAAGTTTTGAC-3′ (forward) and 5’-GCAGCTCTACTCCAGTCTTGCC-3′ (reverse). The value of *SRY* gene expression was normalized to the *GAPDH* expression level and was defined at 1.0.

### BMSC induce Gr-1^Low^CD11b^+^ cells in vitro

A total of 5 × 10^4^ Gr-1^+^CD11b^+^ cells isolated from spleen of naïve C57BL/6 mice by FACS were cultured in RPMI 1640 medium, alone or cocultured with 1 × 10^4^ NIH-3 T3 cells or syngeneic BMSC. Instead of mouse BMSC, some experiments were performed with human BMSC. The concentration of M-CSF in supernatant was detected with a ELISA kit (RayBiotech) according to the manufacturer’s instructions. Transwell studies were performed using 24-well transwell inserts (0.4 μm pores; BD Falcon, San Jose, CA, USA) with BMSC cultured on the culture plates below and Gr-1^+^CD11b^+^ cultured in the inserts. To determine the effect of M-CSF on the differentiation of Gr-1^+^CD11b^+^, recombinant mouse M-CSF (R&D Systems) (1, 5, and 10 ng/mL) was added to Gr-1^+^CD11b^+^ cells (5 × 10^4^ cells/well) isolated from spleen of naïve C57BL/6 mice. Furthermore, Gr-1^+^CD11b^+^ cells isolated from spleen of naïve C57BL/6 mice were cocultured with BMSC transfected with either control siRNA or siM-CSF. siRNAs specific for M-CSF were purchased from Gibco Invitrogen (Waltham, MA, USA). The sequence of s siM-CSF is as follows: GATCCGCAGCAGTTTCATGACCACTTCAAGAGAGTGGTCATGAAACTGCTGCTT. The efficiency of siM-CSF knockdown of BMSC-secreted M-CSF was verified by ELISA (Additional file 2: Figure S2). A total of 24, 48, and 72 h after culture, floating cells were gently collected and numerated using a TC10 automated cell counter (Bio-Rad). The percentage of Gr-1^High^CD11b^+^, Gr-1^High^CD11b^+^ and Gr-1^Low^CD11b^+^ cells was analyzed by FCM and the absolute number of these cells was calculated according to the following formula: Absolute number of Gr-1^High^CD11b^+^ cells = total number of cells harvested from each well × percentage of Gr-1^High^CD11b^+^ (%).

### Statistical analysis

IBM SPSS 23.0 software (IBM Corp, Armonk, NY, USA) was used for statistical analysis. The data were presented as mean ± standard deviation (SD). Statistical analysis was performed using one-way ANOVA for continuous variables. ANOVA was combined with a least significant difference (LSD) to detect which group different from each other. A *p* value < 0.05 was considered statistically significant.

## Results

### BMSC attenuate bleomycin-induced lung injury/fibrosis

To quantitatively assess the degree of pulmonary edema following BLM treatment, the wet/dry weight ratio of the left lung was measured. The BLM-treated mice had a significantly higher wet/dry weight ratio compared with the sham-treated mice; however, BMSC transfer significantly decreased the ratio in the BLM-treated mice (Additional file 3: Figure S3a). Histologic analysis using H&E staining showed exudative change and heavy infiltration of inflammatory cells into the intra-alveolar and interstitial spaces following BLM treatment (Additional file 3: Figure S3b). On day 7, H&E staining of lung sections from BMSC-treated mice had less injury compared with BLM-treated mice. To analyze collagen deposition in lungs, Masson’s trichrome staining was applied to lung tissue sections. The transfer of BMSC nearly abrogated the deposition of collagen on day 28 (Additional file 3: Figure S3b). These histologic results were confirmed by an analysis of collagen in lung tissue lysates by Sircol collagen assay. The total amount of collagen was doubled on day 7 in BLM-treated mice, and was then maintained at the same level up to day 28 (Additional file 3: Figure S3c). The transfer of BMSC reduced the BLM-induced increase in the amount of collagen although this ameliorative effect was incomplete as compared with the sham-treated mice (Additional file 3: Figure S3c).

The levels of IL-1β, VEGF, TGF-β, IL-6, and TNF-α in the BAL fluid of the BLM-treated mice peaked at day 3 to 7, and then decreased slightly up to day 28 (Additional file 4: Figure S4). All values were significantly elevated compared with those in the sham-treated mice. The transfer of BMSC suppressed the increase in IL-1β, TGF-β, and IL-6 after BLM treatment to the level in the sham-treated mice while the suppressive effect was incomplete in VEGF and TNF-α on day 3.

### BMSC tune the differentiation of MDSC in BLM-treated mice

**Fig. 1:**
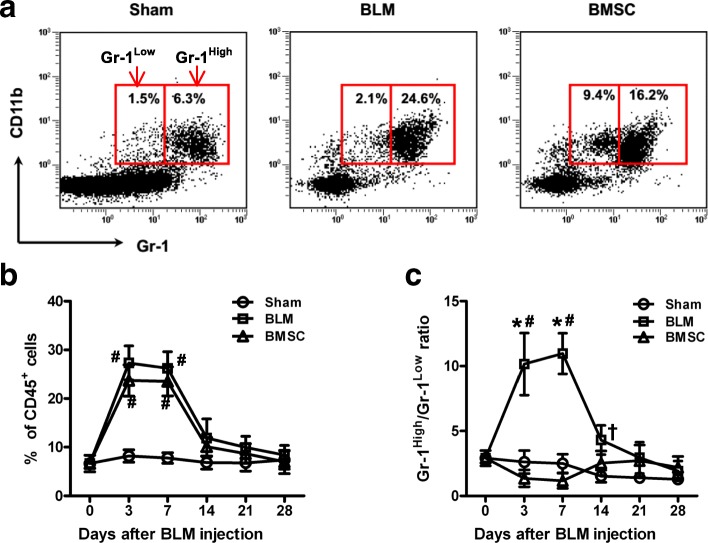
BMSC induced Gr-1^Low^CD11b^+^ cells in BLM-treated mice. C57BL/6 mice were divided into three groups: sham, BLM, and BMSC. Mice in the sham group were injected intratracheally (i.t.) with 30 μL normal saline; the others were injected i.t. with 5 mg/kg BLM in 30 μL normal saline. Twenty-four hours after BLM treatment, 0.1 mL normal saline (BLM group) or BMSC suspension (1 × 10^7^/mL) (BMSC group) was injected through the tail vein. The day of intratracheal injection with BLM or saline was designated as day 0. On days 0, 3, 7, 14, 21, and 28, six mice from each group were euthanized. **a** The proportion of Gr-1^+^CD11b^+^ cells accumulated in the right lungs on day 7 was analyzed by flow cytometry (FCM). Representative FCM data are shown. **b-c** Variations of (**b**) Gr-1^+^CD11b^+^ cells and (**c**) Gr-1^High^CD11b^+^/Gr-1^Low^CD11b^+^ in lungs. Data presented are representative of three replicated experiments. *P < 0.01 as compared with BMSC group; #*P* < 0.01 and †*P* < 0.05 as compared with the sham group. *BLM* bleomycin, *BMSC* bone marrow mesenchymal stem cells

We then sought to characterize the kinetics of the frequencies of Gr-1^+^CD11b^+^ cells. The frequency of Gr-1^+^CD11b^+^cells in the lungs of BLM- and BMSC- treated mice peaked at day 3 then steadily was higher compared with the sham-treated mice throughout the time course (Fig. 1a-b). In sham-treated mice, Gr-1^Low^CD11b^+^ cells were largely absent in the lungs (Fig. 1a). The number of Gr-1^High^CD11b^+^ cells dramatically increased in the lungs while the Gr-1^Low^CD11b^+^ cells slightly increased and therefore the Gr-1^High^/Gr-1^Low^ ratio significantly increased in BLM-treated mice from day 3 to 21 compared with sham-treated mice (Fig. 1c). Transfer of BMSC significantly increased the frequency of Gr-1^Low^CD11b^+^ cells in the lungs of BLM-treated mice. As the Gr-1^Low^CD11b^+^ cells accumulated, the frequencies of Gr-1^High^CD11b^+^ cells decreased and therefore Gr-1^High^/Gr-1^Low^ ratio decreased from day 3 to 14 compared with BLM-treated mice. Thus, the administration of BMSC tunes the differentiation of Gr-1^High^CD11b^+^ toward Gr-1^Low^CD11b^+^ cells.

### Phenotype and morphology of Gr-1^+^CD11b^+^ cells

**Fig. 2:**
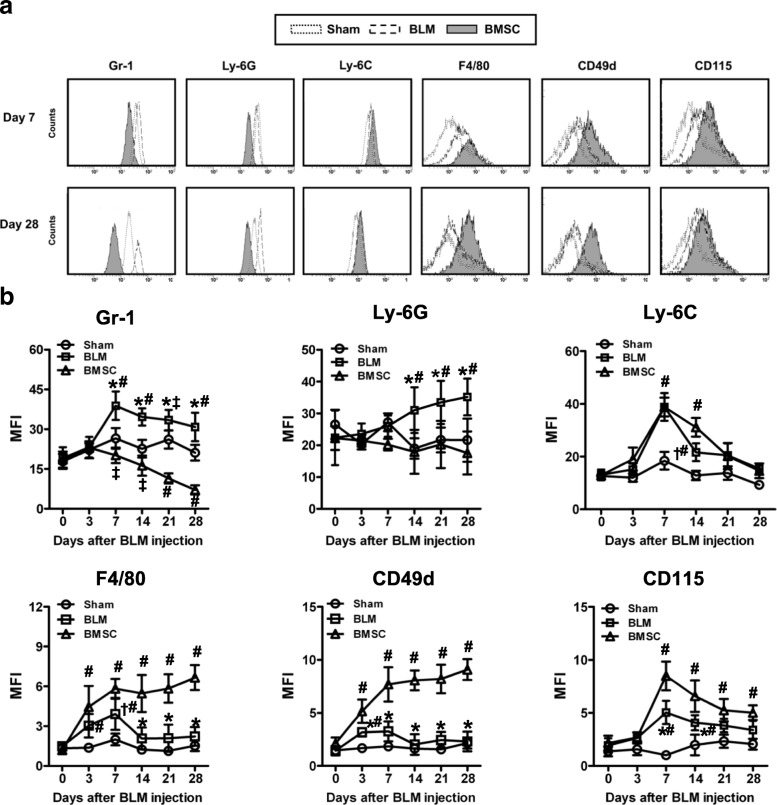
Phenotype of Gr-1^+^CD11b^+^ cells. After BMSC treatment in BLM-treated mice, Gr-1^+^CD11b^+^ cells in the right lungs of sham-, BLM-, and BMSC-treated mice were analyzed for expression of various surface markers. **a** Representative flow cytometry data on day 7 and 28. **b** Expression of indicated markers by Gr-1^+^CD11b^+^ cells in the lungs of sham-, BLM-, and BMSC-treated mice (six mice per group). Data presented are representative of three replicated experiments. *P < 0.01 and †*P* < 0.05 as compared with the BMSC group; and #*P* < 0.01 and ‡*P* < 0.05 as compared with the sham group. *BLM* bleomycin, *BMSC* bone marrow mesenchymal stem cells

To characterize Gr-1 + CD11b + cells, we examined the expression of markers that are known to be expressed by neutrophils and monocytes. In BLM-treated mice, Gr-1 + CD11b + cells expressed high levels of neutrophil-specific marker Gr-1 and Ly-6G and low levels of F4/80, CD49d (α4 integrin, expression of which has been associated with M-MDSC) and CD115 (M-CSF receptor) (Fig. 2a-b). In contrast, Gr-1 + CD11b + cells in BMSC-treated mice expressed high levels of F4/80, CD49d, and CD115, and low levels of Gr-1 and Ly-6G. All Gr-1 + CD11b + cells in both BLM- and BMSC-treated mice were positive for Ly-6C. We also analyzed the phenotype of Gr-1^High^ or Gr-1^Low^ subsets in BMSC-treated mice (Additional file 5: Figure S5a). Gr-1^Low^ cells had lower expression of Gr-1 and Ly-6G, and higher expression of F4/80 and CD49d compared with Gr-1^High^ cells (Additional file 5: Figure S5b). All Gr-1^High^CD11b^+^ and Gr-1^Low^CD11b^+^ cells were positive for Ly-6C. CD115 was also found on some but not all Gr-1^High^CD11b^+^ and Gr-1^Low^CD11b^+^ cells. Overall, Gr-1^High^CD11b^+^ cells expressed markers of neutrophils, whereas Gr-1^Low^CD11b^+^ cells coexpressed neutrophilic and monocytic markers.

We further isolated Gr-1^High^CD11b^+^ and Gr-1^Low^CD11b^+^cells from lungs of BMSC-treated mice and analyzed their nuclear morphology. The majority of Gr-1^High^CD11b^+^ cells had segmented nuclei with a wide cytoplasmic center exhibited characteristics of granulocytic-like cells (Additional file 5: Figure S5c). In contrast, Gr-1^Low^CD11b^+^ cells were composed of mononuclear (MNC)-type cells: the cells had bean-shaped nuclei with a small cytoplasmic center, a characteristic for mononuclear cells. Thus, in both phenotypic and morphological analyses, Gr-1^Low^CD11b^+^ cells appeared as immature myeloid cells, largely of MNC type.

### Pro-inflammatory and immunosuppressive property of Gr-1^+^CD11b^+^ cells

**Fig. 3:**
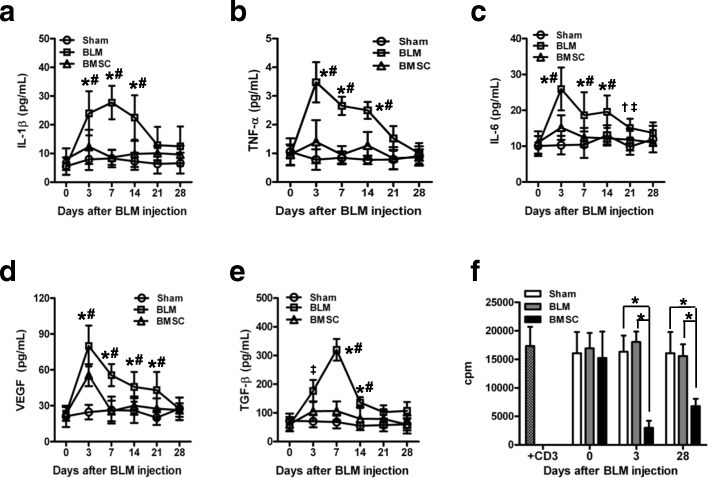
Pro-inflammatory and immunosuppressive property of Gr-1^+^CD11b^+^ cells. **a-e** After BMSC treatment in BLM-treated mice, Gr-1^+^CD11b^+^ cells in the lungs of sham-, BLM- and BMSC-treated mice (three mice per group) were isolated and cultured in triplicate (1 × 10^5^ cells/well). Twenty-four hours later, supernatants were collected and the levels of IL-1β, VEGF, TGF-β, IL-6, and TNF-α were measured using ELISA. *P < 0.01 and †*P* < 0.05 as compared with the BMSC group; and #*P* < 0.01, and ‡*P* < 0.05 as compared with the sham group. **f** CD3^+^ cells (2 × 10^5^ cells/well) isolated from spleens of naïve C57BL/6 mice were stimulated with CD3-specific antibodies (1 μg/mL) in the presence of Gr-1^+^CD11b^+^ cells (1 × 10^5^ cells/well) sorted from the lungs of sham-, BLM-, and BMSC-treated mice (three mice per group). T cell proliferation was measured in triplicate by ^3^H-thymidine incorporation. Data presented are representative of three replicated experiments. *P < 0.01. *BLM*, bleomycin, *BMSC* bone marrow mesenchymal stem cells

To more precisely characterize Gr-1^+^CD11b^+^ cells, we examined the expression of pro-inflammatory cytokines that are known to be pathogenic in BLM-induced lung injury. The concentration of IL-1β, TNF-α, IL-6, VEGF, and TGF-β secreted from Gr-1^+^CD11b^+^ cells isolated from the lungs of BLM-treated mice was significantly increased as compared with sham-treated mice throughout the time course (Fig. 3a-e). In contrast, BMSC transfer abrogated the increase of IL-1β, TNF-α, IL-6, VEGF, and TGF-β in BLM-treated mice (Fig. 3a-e). To evaluate the pro-inflammatory activity of Gr-1^High^ and Gr-1^Low^ cells, we examined the concentration of pro-inflammatory cytokines in the supernatants of cultured Gr-1^High^ and Gr-1^Low^ cells isolated from lungs of BMSC-treated mice. The concentration of IL-1β and TNF-α in Gr-1^High^CD11b^+^ was significantly increased compared with Gr-1^Low^CD11b^+^ cells (Additional file 6: Figure S6a-b). In contrast, Gr-1^Low^CD11b^+^ produced more IL-6 and VEGF than Gr-1^High^CD11b^+^ subset (Additional file 6: Figure S6c-d). No statistical differences were observed in the level of TGF-β between those cell populations (Additional file 6: Figure S6e).

The immunosuppressive activity of Gr-1^+^CD11b^+^ cells from lungs of BLM- and BMSC-treated mice were also evaluated on their ability to inhibit CD3-inducible T cell proliferation. On day 3, no inhibition of T cell proliferation was observed in Gr-1^+^CD11b^+^ cells isolated from BLM-treated mice (Fig. 3f). However, Gr-1^+^CD11b^+^ cells isolated from BMSC-treated mice had potent immunosuppressive activity. Meanwhile, on day 28 after BLM exposure, significant inhibition of T cell proliferation was also observed in BMSC-treated mice but not in BLM-treated mice. The immunosuppressive activity of Gr-1^Low^CD11b^+^ and Gr-1^High^CD11b^+^ cells sorted from lungs of BMSC-treated mice was further evaluated. On day 3 and 28 after BLM exposure, Gr-1^High^CD11b^+^ cells did not inhibit T cell proliferation while the Gr-1^Low^CD11b^+^ showed significant immunosuppressive activity (Additional file 6: Figure S6f). These results suggest that Gr-1^Low^ cells produce less pro-inflammatory cytokines and obtain immunosuppressive activity that may ameliorate BLM-induced pulmonary injury.

### BMSC-derived M-CSF promotes Gr-1^Low^CD11b^+^ expansion

**Fig. 4:**
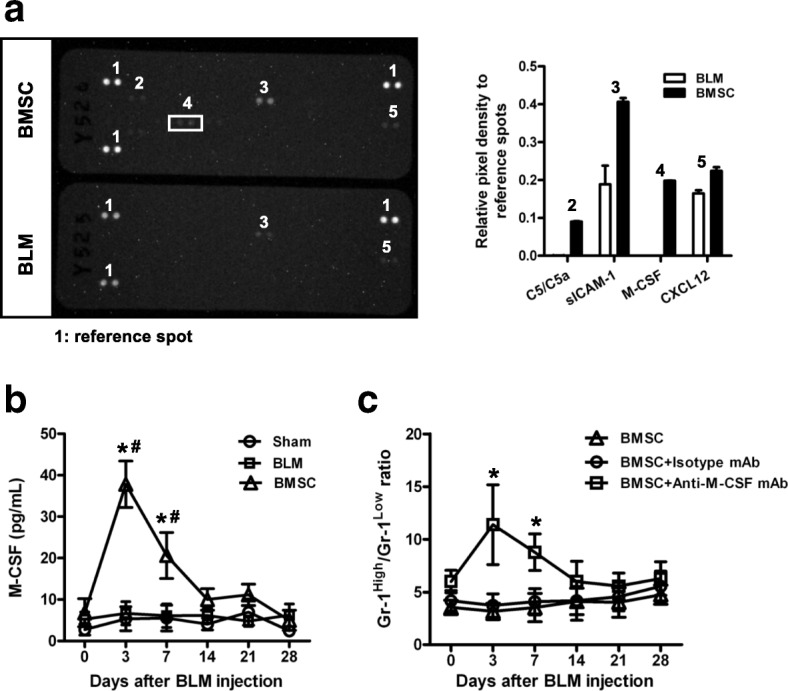
BMSC expansion of Gr-1^Low^CD11b^+^ is mediated by M-CSF. Twenty-four hours after BMSC treatment in BLM-treated mice, sera were collected from BLM and BMSC-treated mice (six mice from each group). **a** The relative expression levels of 40 mouse soluble proteins in the sera of BLM- and BMSC-treated mice were measured in duplicate using a Mouse Cytokine Array. The results shown are normalized to reference spots. **b** The concentration of M-CSF in serum collected from sham-, BLM-, and BMSC-treated mice (six mice from each group) was measured with ELISA in triplicate. Data presented are representative of two replicated experiments. *P < 0.01 as compared with the BLM group; #*P* < 0.01 as compared with the sham group. **c** C57BL/6 mice were treated with 5 mg/kg BLM in 30 μL normal saline via intratracheal instillation. BMSC (1 × 10^6^/mouse) were administered via the tail vein 24 h after BLM treatment. On day 0, 2, 4, and 6 after BMSC administration, these mice were treated with 10 μg of anti-M-CSF mAb or isotype mAb i.p.. Lung specimens (six lungs from each group) were collected and the variations of Gr-1^High^CD11b^+^/Gr-1^Low^CD11b^+^ were analyzed. *P < 0.01 as compared with the BMSC and the BMSC+Isotype mAb group. *BLM* bleomycin, *BMSC* bone marrow mesenchymal stem cells

Next, we investigated which soluble factors were responsible for the observed modulatory effect of BMSC on the differentiation of Gr-1^+^CD11b^+^ cells. The analysis of soluble parameters in BMSC-treated mice showed an increase of C5/C5a, sICAM-1, M-CSF, and CXCL-12 as compared with BLM-treated mice (Fig. 4a). M-CSF is a hematopoietic growth factor that is involved in the proliferation, differentiation, and survival of monocytes, macrophages, and bone marrow progenitor cells [25]. Therefore, we tested the serum level of M-CSF, and it was very clear that the concentration of M-CSF significantly increased in BMSC-treated mice compared with sham- and BLM-treated mice (Fig. 4b). To confirm if M-CSF is the key factor for the expansion of Gr-1^Low^CD11b^+^ in BMSC-treated mice, we neutralized M-CSF in vivo after BMSC transfer. The results showed that neutralization of M-CSF abrogated the expansion of Gr-1^Low^CD11b^+^ cells after BMSC therapy (Fig. 4c). Collectively, these data indicated that expansion of Gr-1^Low^CD11b^+^ cells by BMSC is mediated by M-CSF.

### Engraftment of donor-derived BMSC in BLM-treated mice

**Fig. 5:**
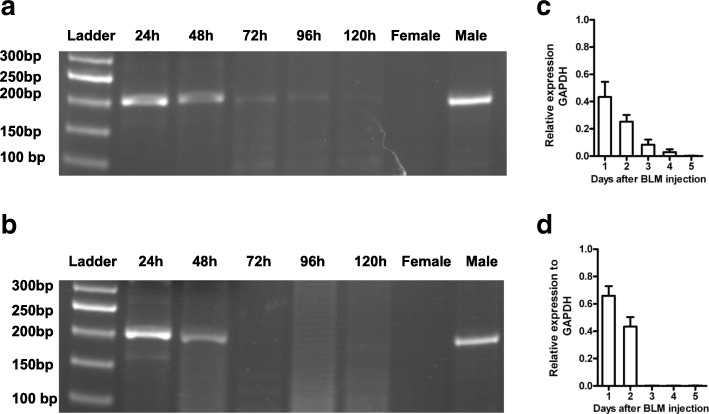
Detection of allogeneic BMSC in lungs and peripheral blood of BLM-treated mice. After male BMSC treatment in the BLM-treated female mice, peripheral blood and lung samples were collected at different time points. **a-b** Representative PCR analysis of *SRY* gene from (**a**) lung and (**b**) peripheral blood from BLM-treated female mice transplanted with male BMSC is shown. For PCR, peripheral blood and lung samples collected from female and male mice were used as negative and positive control. **c** Lung specimens and (**d**) peripheral blood were collected and the mRNA expression of *SRY* gene was assessed by quantitative RT-PCR (*n* = 3). The results shown are normalized to GAPDH

To demonstrate the localization of the infused BMSC in the host, PCR using a mouse Y chromosome primer was performed to identify the engraftment of the male donor cells in the lungs and peripheral blood of BMSC-treated mice. As showed in Fig. 5a, *SRY* DNA was detectable until 96 h in the lungs of mice that had received i.v. BMSC transfer. Donor-derived *SRY* DNA was detected at 48 h in the peripheral blood of BLM-treated mice (Fig. 5b). Quantitative RT-PCR detection showed that mice injured with BLM and treated with BMSC exhibited mRNA expression of *SRY* gene within 96 and 48 h in lung tissues and peripheral blood respectively (Fig. 5c-d).

### BMSC tune the differentiation of MDSC in vitro

**Fig. 6:**
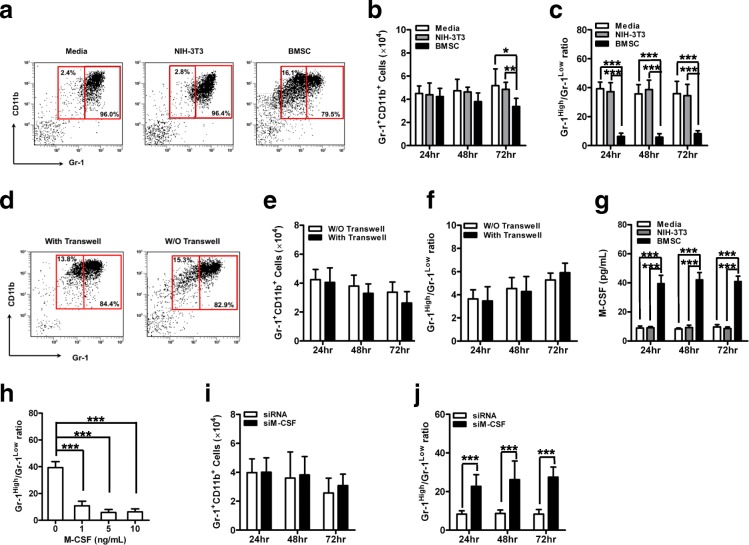
BMSC tune the differentiation of MDSC in vitro. Gr-1^+^CD11b^+^ cells (5 × 10^4^ cells/well) isolated from spleens of naïve C57BL/6 mice (n = 3) were cultured alone or in the presence of NIH-3 T3 or BMSC (1 × 10^4^ cells/well). A total of 24, 48, and 72 h after coculture, floating cells were collected and numerated. **a** Seventy-two hours after coculture, the phenotype of floating cells was analyzed by flow cytometry (FCM). Representative FCM data are shown. **b** The absolute number of Gr-1^+^CD11b^+^ cells and (**c**) the variations of Gr-1^High^CD11b^+^/Gr-1^Low^CD11b^+^ were calculated. **d-f** BMSC and Gr-1^+^CD11b^+^ cells were co-cultured and transwell inserts were used in some wells to prevent cell-cell contact. **d** Seventy-two hours after coculture, the phenotype of floating cells was analyzed by flow cytometry (FCM). Representative FCM data are shown. **e** The absolute number of Gr-1^+^CD11b^+^ cells and (**f**) the variations of Gr-1^High^CD11b^+^/Gr-1^Low^CD11b^+^ were calculated. **g** The concentration of M-CSF was measured in the supernatant using ELISA. **h** Gr-1^+^CD11b^+^ cells isolated from spleen of naïve C57BL/6 mice were cultured with exogenous recombinant mouse M-CSF. The variations of Gr-1^High^CD11b^+^/Gr-1^Low^CD11b^+^ were calculated. **i-j** Gr-1^+^CD11b^+^ cells isolated from spleens of naïve C57BL/6 mice were cocultured with BMSC transfected with either control siRNA or siM-CSF (n = 3). **i** The absolute number of Gr-1^+^CD11b^+^ cells and (**j**) the variations of Gr-1^High^CD11b^+^/Gr-1^Low^CD11b^+^ were calculated. Data presented are representative of two replicated experiments. *P < 0.05, **P < 0.01 and ***P < 0.001. *BMSC* bone marrow mesenchymal stem cells, *mAb* monoclonal antibody, *W/O* without

To confirm the in vivo modulation of Gr-1^+^CD11b^+^ cells by BMSC, we performed a series of Gr-1^+^CD11b^+^ cells/BMSC coculture experiments in vitro. First, the addition of BMSC to Gr-1^+^CD11b^+^ cells caused reduction of absolute Gr-1^+^CD11b^+^ cell number after 72 h coculture (Fig. 6a-b). In particular, Gr-1^Low^ was significantly higher in the Gr-1^+^CD11b^+^ cells/BMSC coculture system and consequently, Gr-1^High^/Gr-1^Low^ ratio decreased compared with Gr-1^+^CD11b^+^ cells alone or the NIH3T3/BMSC coculture group (Fig. 6c). Furthermore, human BMSC exerted similar effects on the differentiation of mouse Gr-1^+^CD11b^+^ cells in vitro (Additional file 7: Figure S7). Next, the induction of Gr-1^Low^ cells was independent of cell-cell contact when BMSC and Gr-1^+^CD11b^+^ cells were separated in a transwell experiment (Fig. 6d-f). We further investigated whether the M-CSF could be a key factor involved in Gr-1^Low^ mobilization in vitro as well as in vivo. An ELISA test revealed that the concentration of M-CSF from the Gr-1^+^CD11b^+^ cells/BMSC coculture group significantly increased compared with Gr-1^+^CD11b^+^ cells alone or NIH3T3/BMSC coculture group (Fig. 6g). We found that the addition of recombinant M-CSF alone could tune the differentiation of Gr-1^+^CD11b^+^ cells toward Gr-1^Low^CD11b^+^ (Fig. 6h). When Gr-1^+^CD11b^+^ cells were cocultured with BMSC silenced for M-CSF expression, there was a significant increase in Gr-1^High^/Gr-1^Low^ ratio, while the Gr-1^+^CD11b^+^ cell number was not influenced (Fig. 6i-j). In total, BMSC mediate the expansion of Gr-1^Low^CD11b^+^ subset by M-CSF, which tunes the differentiation of Gr-1^High^CD11b^+^ toward the Gr-1^Low^CD11b^+^ subset.

## Discussion

In this study, we found that in lungs of BLM-treated mice were of high abundance of granulocytic Gr-1^High^CD11^+^ cells and consequently these cells may be a predominant pathogenic factor in BLM-induced lung injury via releasing pro-inflammatory cytokines. The downregulation of Gr-1^High^CD11^+^ cells in BMSC-treated mice accompanied with increase of Gr-1^Low^CD11^+^ cells simultaneously that suggested that these myeloid-derived cells were purposefully modulated. Furthermore, this modulation effect was independent of engraft of BMSC in lung tissue, which suggested that BMSC served as a paracrine function in the BLM-induced lung injury model. To the best of our knowledge, this is the first description of MDSC during BLM-induced pulmonary injury.

Previously, several studies have described the increase of neutrophils at the early stage of BLM-treated mice and have associated the accumulation of these cells with the progression of BLM-induced pulmonary injury [17–21]. However, most of the studies did not take into account the levels of Gr-1 expression and considered all Gr-1^+^ cells as neutrophils. Our study demonstrates that Gr-1^High^ cells that accumulate abundantly at the early stages of BLM-treated mice are similar to typical neutrophils. This is supported by cell phenotype, nuclear morphology, and pro-inflammatory but not immunosuppressive characteristics. Our data on accumulation of pro-inflammatory Gr-1^High^CD11b^+^ cells was consistent with some reports on the role for neutrophils in BLM-induced lung injury and fibrosis. Indeed, in the early stage of BLM-induced lung injury, high counts of neutrophils and high levels of neutrophil-derived pro-inflammatory cytokines and elastase were associated with high risk of lung injury and fibrosis progression [17–21, 26–28], suggesting a significant role for neutrophils in lung injury and fibrosis development. On the other hand, Ortiz  et al. have demonstrated that Gr-1^+^CD11b^+^ cells accumulate abundantly in the lungs of mice susceptible to cigarette smoke and these cells were largely represented by the granulocytic MDSC [29]. However, these cells lacked immunosuppressive activity, indicating that they were not bona fide MDSC. Immunosuppressive MDSC accumulated in these mice only after the development of tumor lesions. Our findings extend those of Ortiz , demonstrating that BLM-induced injury, despite causing the expansion of Gr-1^High^CD11b^+^ cells, was not sufficient to convert them into canonical MDSC. These results were consistent with the two-signal concept of MDSC accumulation in cancer, suggesting that the expansion of myeloid cells and their conversion to MDSC phenotype was governed by different factors and signaling pathways [30]. The first group of signals is important for the expansion of populations of immature myeloid cells, whereas the second group is responsible for their pathologic activation [10, 30]. Some factors involved in the expansion of MDSC are well characterized [i.e., granulocyte macrophage colony-stimulating factor (GM-CSF), M-CSF]. However, the mechanism responsible for MDSC conversion is largely unclear and needs to be determined.

MSC and MDSC belong to distinct differentiation lineages; however, their immunoregulatory properties have several common traits [31]. Only few studies directly addressed the interplay between MSC and MDSC. In the study by Yen et al., human MSC expanded CD11b^+^CD33^+^CD14^―^ MDSC that expressed arginase 1 (ARG1) and nitric oxide (NO), suppressed T cell proliferation, and promoted regulatory T cell generation [32]. The effect was mediated through the secretion of hepatic growth factor (HGF) and the induction of signal transducers and activators of transcription 3 (STAT3). We have demonstrated that following systemic administration, BMSC inhibited the activation and proliferation of MDSC and further prevented tumor metastasis formation in a mouse model [33, 34]. This regulatory property was further defined using an in vitro MDSC-inducing system, which demonstrated significant suppressive effect of BMSC on the MDSC proliferation. However, the mechanisms involved in regulatory effects of BMSC on MDSC remain unknown. In this study, we have demonstrated that BMSC tune the differentiation of inflammatory Gr-1^High^CD11b^+^ toward immunosuppressive Gr-1^Low^CD11b^+^ subsets in the BLM-induced lung injury. Phenotypic, morphological, and functional analyses of Gr-1^Low^CD11b^+^ cells characterized them as immature myeloid-derived suppressor cells: (a) the cells coexpressed neutrophilic (Gr-1 and Ly-6G) and monocytic (F4/80 and CD49d) markers; (b) the cells had nuclei of immature monocytes/granulocytes and myelomonocytic precursors; and (c) the cells were able to suppress T cell proliferation in vitro. This is the first report, to our knowledge, that demonstrates that BMSC directly tune the differentiation of inflammatory Gr-1^High^CD11b^+^ toward immunosuppressive Gr-1^Low^CD11b^+^ subsets and contributes to the immunomodulatory properties of BMSC.

Results obtained in our study raise several questions. One of the questions is which factors induce Gr-1^Low^CD11b^+^cells during BMSC treatment. Studies performed in other pathological conditions (e.g., tumors) suggest that the main factors that induce MDSC generation are growth factors and pro-inflammatory cytokines (i.e., GM-CSF, M-CSF) [10, 11]. It has been documented that MSCs block the differentiation of monocytes and CD34^+^ progenitors into CD1a^+^ DCs and redirect their differentiation toward the immature myeloid cells, which are partially mediated by soluble factors, such as M-CSF and IL-6 [35]. Consistent with these results, our data suggest that through M-CSF and the consequent expansion of monocytic Gr-1^Low^CD11b^+^ cells, BMSC may play a role in MDSC differentiation. Our findings can help to explain the strong association of M-CSF secreted by BMSC with MDSC, since it is well established that in the acute stress situation, M-CSF is highly secreted by bone marrow stromal cells, promoting proliferation, differentiation, and survival of blood monocytes and their progenitor cells [36]. While M-CSF has been implicated in immunoregulatory responses, and the BMSC, which naturally secretes M-CSF, was reported to have some immunological functions upon MDSC [33, 34], the specific molecular mechanisms underlying these observations have been largely unexplored. Gr-1^+^CD11b^+^ cells expressing M-CSF receptor (CD115) and CD49d have been shown to have immunosuppressive activity [12, 37, 38]. We found higher levels of CD115 and CD49d expression in monocytic Gr-1^Low^CD11b^+^ cells in BMSC-treated mice. After sorting of these cells, we found that CD115 and CD49d-positive MDSC suppressed T cell proliferation compared with granulocytic Gr-1^High^CD11b^+^ cells. Our data support previous observation that MDSC-expressing M-CSF receptor has potent immune suppressive activity.

There are many studies indicating the homing ability of bone marrow cells to injured lung and active differentiation into many types of cells [18, 39, 40]. In contrast, two recent studies failed to observe bone marrow transplantation-induced lung reconstitution in transgenic mice infused with surfactant protein C-enhanced green fluorescent protein transgenic bone marrow cells [41, 42]. Furthermore, accumulating evidence suggests that BMSC induced tissue protection is provided not by donor cell replacement of damaged lung cells, but rather by humoral factors released from the injected cells, such as growth factors and anti-inflammatory cytokines [19, 43–46]. Therefore, when considered that the donor-derived cells were sparsely detected from day 2 to 4 and the overall engraftment level were very low through the entire experimental period, the paracrine release of growth factors and anti-inflammatory cytokines by, or induced by, BMSC may be important mediators of tissue repair.

## Conclusions

In conclusion, our study demonstrates that Gr-1^High^CD11b^+^ cells abundantly accumulate at early stage of BLM-induced lung injury, and possess pro-inflammatory properties able to induce lung injury. The systemic administration of BMSC effectively tunes the differentiation of Gr-1^High^CD11b^+^ toward Gr-1^Low^CD11b^+^ cells, which possess immunosuppressive activity and may ameliorate lung injury and collagen deposition. These data suggest that BMSC transfer may be an effective strategy for the inhibition of progression of lung injury and fibrosis via modulation of the microenvironment of injured lung.

## Additional files

Additional file 1: **Figure S1.** Characterization of mouse BMSC. (PDF 294 kb)

Additional file 2: **Figure S2.** Knockdown of M-CSF expression in mouse BMSC. (PDF 55 kb)

Additional file 3: **Figure S3.** BMSC ameliorated BLM-induced pulmonary edema and fibrosis. (PDF 313 kb)

Additional file 4: **Figure S4.** BMSC reduce the levels of IL-1β, VEGF, TGF-β, IL-6, and TNF-α in bronchoalveolar lavage (BAL) fluid. (PDF 293 kb)

Additional file 5: **Figure S5.** Phenotype and morphology of Gr-1^High^CD11b^+^ and Gr-1^Low^CD11b^+^ cells isolated from lungs of BMSC-treated mice. (PDF 350 kb)

Additional file 6: **Figure S6.** Pro-inflammatory and immunosuppressive property of Gr-1^High^CD11b^+^ and Gr-1^Low^CD11b^+^ cells. (PDF 235 kb)

Additional file 7: **Figure S7.** Human BMSC tune the differentiation of mouse MDSC in vitro. (PDF 100 kb)

## Abbreviations

BAL: Bronchoalveolar lavage
BLM: Bleomycin
BMSC: Bone marrow mesenchymal stem cell
ELISA: Enzyme-linked immunosorbent assay
FACS: Fluorescence-activated cell sorting
FCM: Flow cytometry
IL: Interleukin
mAb: Monoclonal antibody
M-CSF: Macrophage colony-stimulating factor
MDSC: Myeloid-derived suppressor cell
PBS: Phosphate-buffered saline
RT-qPCR: Reverse-transcription quantitative polymerase chain reaction
SRY: sex Determination region of Y chromosome
TGF-β: Transforming growth factor beta
TNF-α: Tumor necrosis factor alpha
VEGF: Vascular epithelial growth factor
