# Supplementary material for: Bone marrow mesenchymal stem cells tune the differentiation of myeloid-derived suppressor cells in bleomycin-induced lung injury
Source: Stem Cell Res Ther. 2018 Sep 26;9:253. doi: 10.1186/s13287-018-0983-1 (PMC6158827; doi:10.1186/s13287-018-0983-1)
Supplement: Supplementary file 1 — Figure S1. Characterization of mouse BMSC. (PDF 294 kb) [file 13287_2018_983_MOESM1_ESM.pdf]

Additional file 1: Figure S1

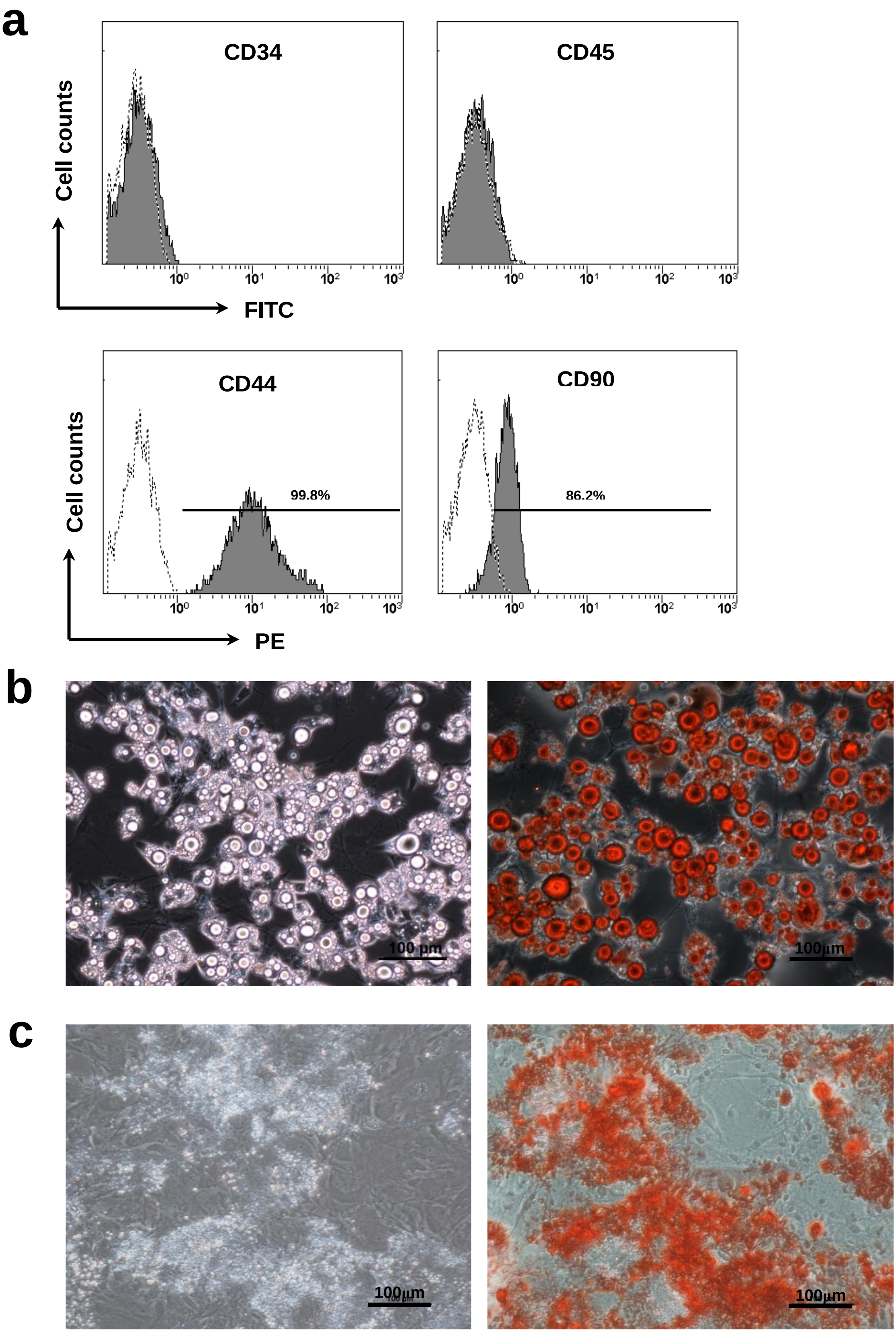

Additional file 1: Figure S1. Characterization of mouse BMSC. a Flow cytometric analysis of BMSC. Dash line: fluorescent-conjugated isotype mAb. Solid line: fluorescent-conjugated anti-CD34, CD44, CD45 and CD90 mAb. b Oil droplet formation in the differentiated adipocytes was observed by light

microscopy (left) and demonstrated by oil red O staining (right). c Calcium deposition in the differentiated osteocytes was observed by light microscopy (left) and showed by alizarin red staining (right).
