# Supplementary material for: Bone marrow mesenchymal stem cells tune the differentiation of myeloid-derived suppressor cells in bleomycin-induced lung injury
Source: Stem Cell Res Ther. 2018 Sep 26;9:253. doi: 10.1186/s13287-018-0983-1 (PMC6158827; doi:10.1186/s13287-018-0983-1)
Supplement: Supplementary file 2 — Figure S2. Knockdown of M-CSF expression in mouse BMSC. (PDF 55 kb) [file 13287_2018_983_MOESM2_ESM.pdf]

Additional file 2: Figure S2

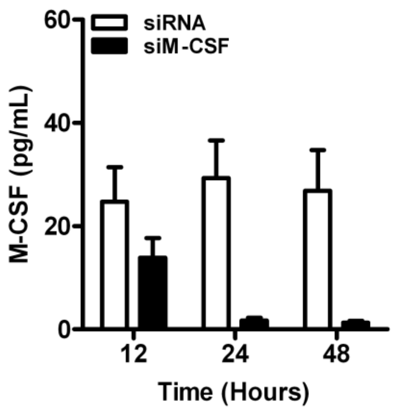

Additional file 2: Figure S2. Knockdown of M-CSF expression in mouse BMSC. BMSC transfected with either control siRNA or siM-CSF (1×10^4 cells/well) were cultured in vitro. The concentration of M-CSF was measured in the supernatant of each well using ELISA at indicated time point.
