# Supplementary material for: Bone marrow mesenchymal stem cells tune the differentiation of myeloid-derived suppressor cells in bleomycin-induced lung injury
Source: Stem Cell Res Ther. 2018 Sep 26;9:253. doi: 10.1186/s13287-018-0983-1 (PMC6158827; doi:10.1186/s13287-018-0983-1)
Supplement: Supplementary file 3 — Figure S3. BMSC ameliorated BLM-induced pulmonary edema and fibrosis. (PDF 313 kb) [file 13287_2018_983_MOESM3_ESM.pdf]

Additional file 3: Figure S3

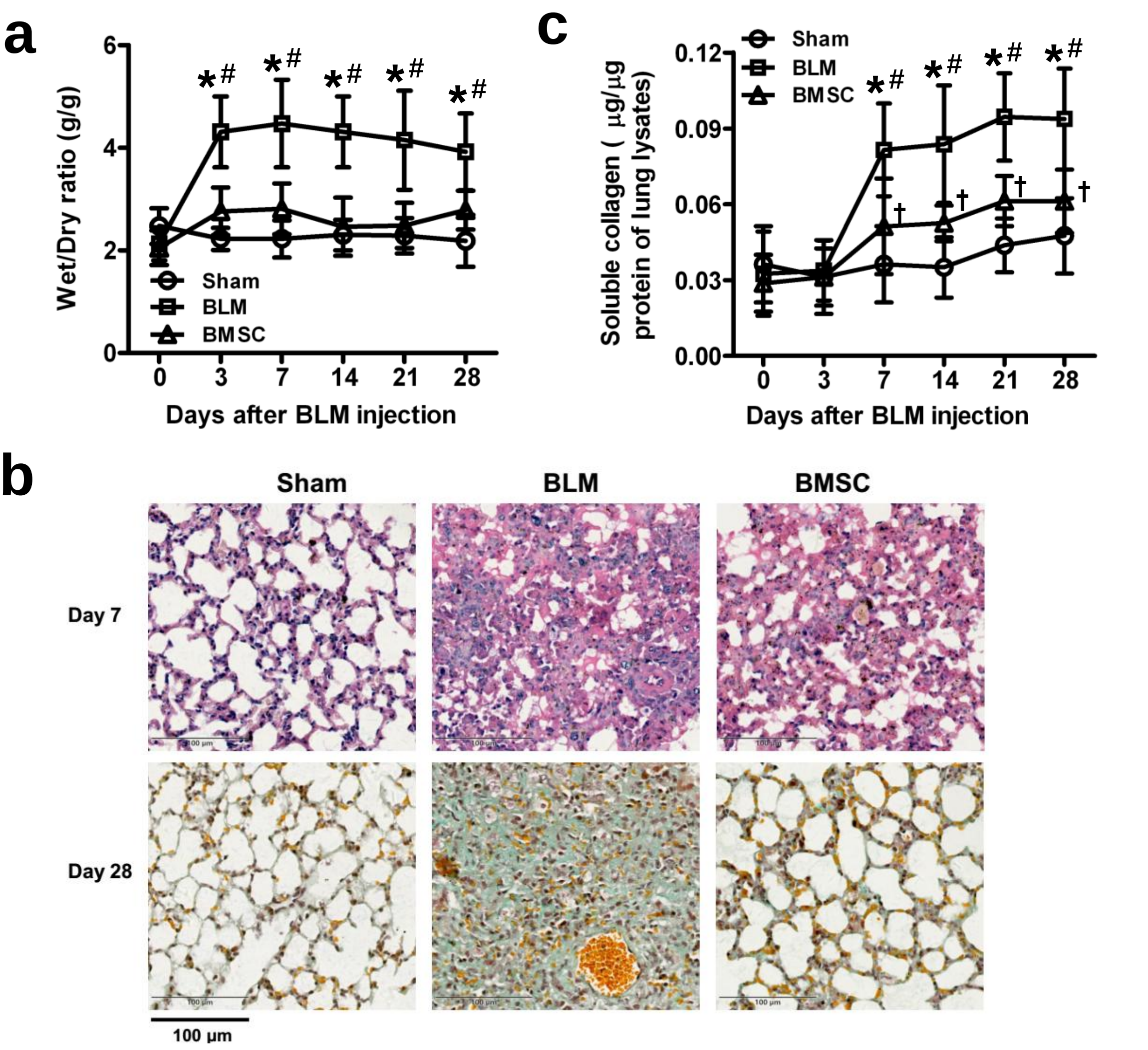

Additional file 3: Figure S3. BMSC ameliorated BLM-induced pulmonary edema and fibrosis. C57BL/6 mice were divided into 3 groups: Sham, BLM and BMSC group. Mice in the sham group were injected intratracheally (i.t.) with 30μL normal saline; the others were injected i.t. with BLM (5 mg/kg in 30μL normal saline). Twenty-four hours after BLM treatment, 0.1mL normal saline (BLM group) or BMSC suspension (1 × 10^7 cells/mL) (BMSC group) was injected through tail vein. The day of intratracheal injection with BLM or saline was designated as Day 0. On days 0, 3, 7, 14, 21, and 28, 6 mice from each group were killed. a Effect of BMSC transfer on the BLM-induced increase in the lung wet/dry ratio. b Effect of BMSC transfer on BLM-induced histologic changes in the lungs. Lung tissues were stained with hematoxylin & eosin (above) or massion (below) on day 7 and 28. c The sircol collagen assay was used to measure the total amount of soluble collagen in lung tissue lysates. Data presented are representative of three replicated experiments. *P < 0.01 as

compared with "BMSC" group; # *P* < 0.01 and † *P* < 0.05 as compared with "Sham" group. BLM, bleomycin; BMSC, bone marrow mesenchymal stem cells.
