# Supplementary material for: Bone marrow mesenchymal stem cells tune the differentiation of myeloid-derived suppressor cells in bleomycin-induced lung injury
Source: Stem Cell Res Ther. 2018 Sep 26;9:253. doi: 10.1186/s13287-018-0983-1 (PMC6158827; doi:10.1186/s13287-018-0983-1)
Supplement: Supplementary file 4 — Figure S4. BMSC reduce the levels of IL-1β, VEGF, TGF-β, IL-6, and TNF-α in bronchoalveolar lavage (BAL) fluid. (PDF 293 kb) [file 13287_2018_983_MOESM4_ESM.pdf]

Additional file 4: Figure S4

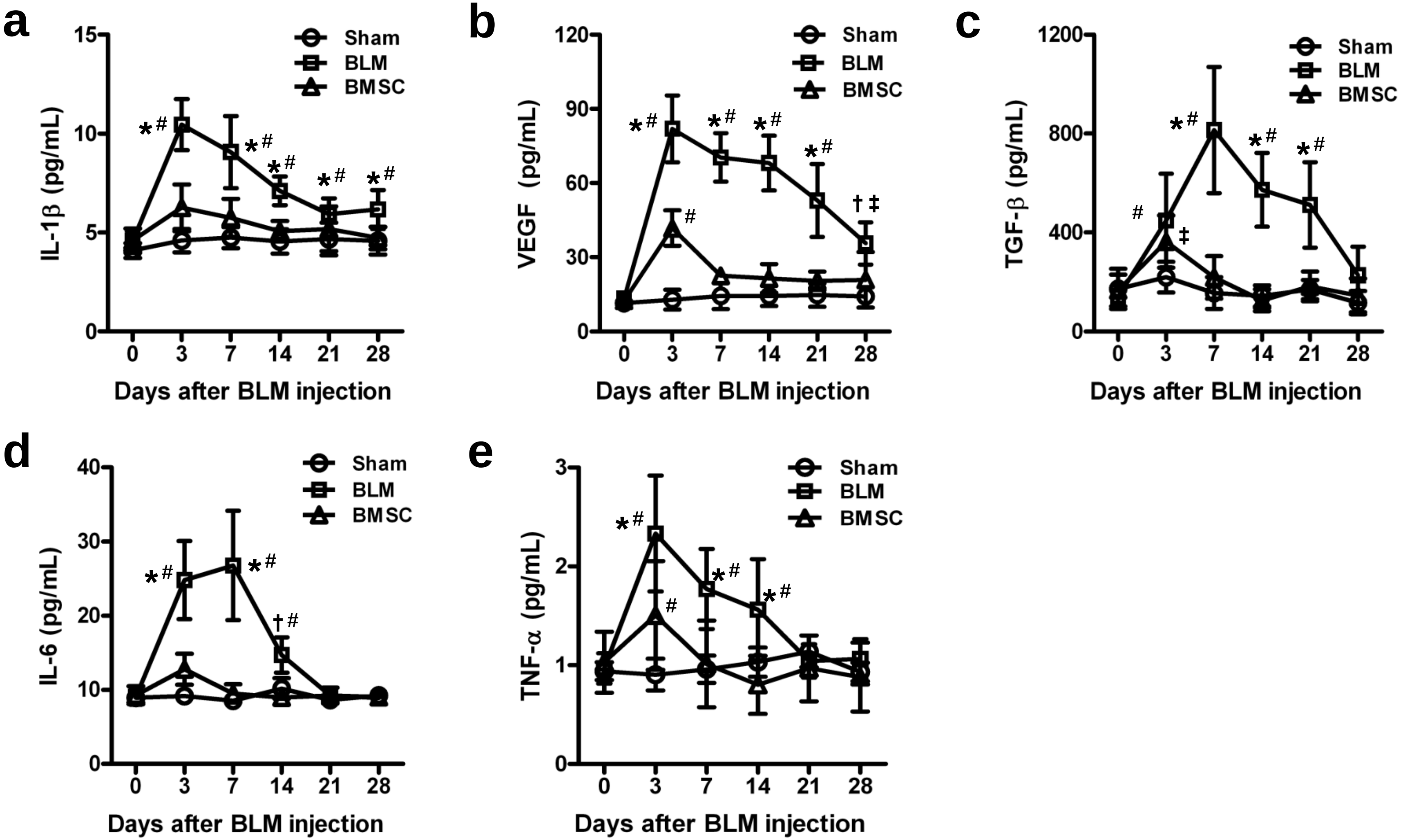

Additional file 4: Figure S4. BMSC reduces the levels of IL-1β, VEGF, TGF-β, IL-6, and TNF-α in bronchoalveolar lavage (BAL) fluid. **After BMSC treatment in BLM-induced lung injury mouse, BAL was flushed from the Sham-, BLM- and BMSC-treated mice (3 mice per group).** The levels of **(a)** IL-1β, **(b)** VEGF, **(c)** TGF-β, **(d)** IL-6 and **(e)** TNF-α were measured by ELISA **using triplicate samples. Data presented are representative of two replicated experiments.** *$P < 0.01$ and † $P < 0.05$ as compared with "BMSC" group; # $P < 0.01$ and ‡ $P < 0.05$ as compared with "Sham" group. BLM, bleomycin; BMSC, bone marrow mesenchymal stem cells; IL: interleukin; TNF-α, tumor necrosis factor-α; TGF-β, transforming growth factor-β; VEGF, vascular epithelial growth factor.
