# Supplementary material for: Bone marrow mesenchymal stem cells tune the differentiation of myeloid-derived suppressor cells in bleomycin-induced lung injury
Source: Stem Cell Res Ther. 2018 Sep 26;9:253. doi: 10.1186/s13287-018-0983-1 (PMC6158827; doi:10.1186/s13287-018-0983-1)
Supplement: Supplementary file 5 — Figure S5. Phenotype and morphology of Gr-1HighCD11b+ and Gr-1LowCD11b+ cells isolated from lungs of BMSC-treated mice. (PDF 350 kb) [file 13287_2018_983_MOESM5_ESM.pdf]

Additional file 5: Figure S5

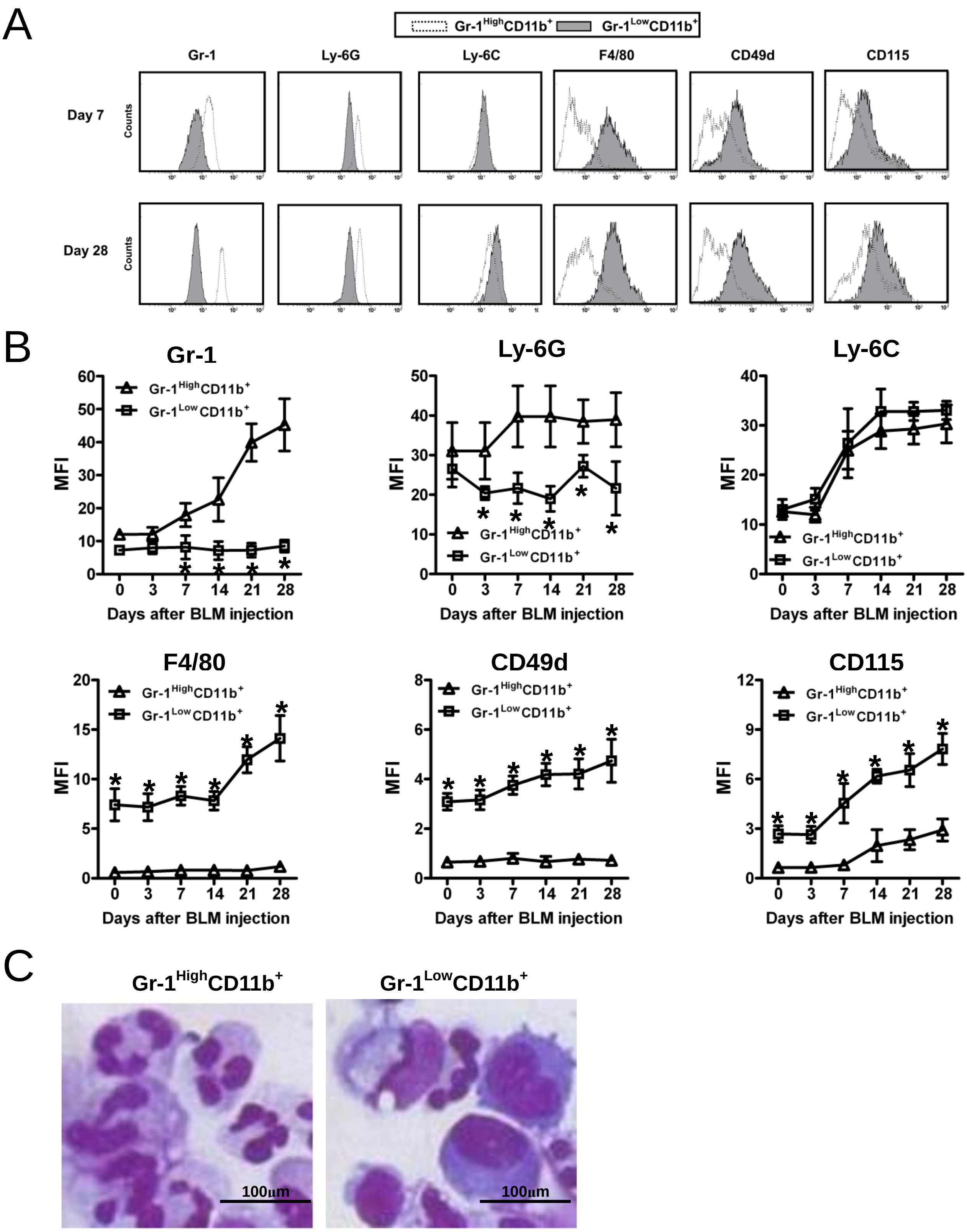


**Additional file 5: Figure S5.** Phenotype and morphology of Gr-1$^{High}$CD11b$^{+}$ and Gr-1$^{Low}$CD11b$^{+}$ cells. **After BMSC treatment in the BLM-treated mice, Gr-1$^{High}$CD11b$^{+}$ and Gr-1$^{Low}$CD11b$^{+}$ cells isolated from lungs of BMSC-treated mice (n=6) were analyzed for expression of various surface markers. a Representative flow cytometry data on day 7 and 28.** **b** Variations of surface markers on Gr-1$^{High}$CD11b$^{+}$ and Gr-1$^{Low}$CD11b$^{+}$ cells in lungs of BMSC-treated mice. **c** Nuclear morphology of Gr-1$^{High}$CD11b$^{+}$ and Gr-1$^{Low}$CD11b$^{+}$ cells in lungs of BMSC-treated mice was demonstrated by Wright staining. *$P < 0.01$ as compared with “Gr-1$^{High}$CD11b$^{+}$” group. BLM, bleomycin; BMSC, bone marrow mesenchymal stem cells.

MFI, mean fluorescence intensity.
