# Supplementary material for: Bone marrow mesenchymal stem cells tune the differentiation of myeloid-derived suppressor cells in bleomycin-induced lung injury
Source: Stem Cell Res Ther. 2018 Sep 26;9:253. doi: 10.1186/s13287-018-0983-1 (PMC6158827; doi:10.1186/s13287-018-0983-1)
Supplement: Supplementary file 6 — Figure S6. Pro-inflammatory and immunosuppressive property of Gr-1HighCD11b+ and Gr-1LowCD11b+ cells. (PDF 235 kb) [file 13287_2018_983_MOESM6_ESM.pdf]

Additional file 6: Figure S6

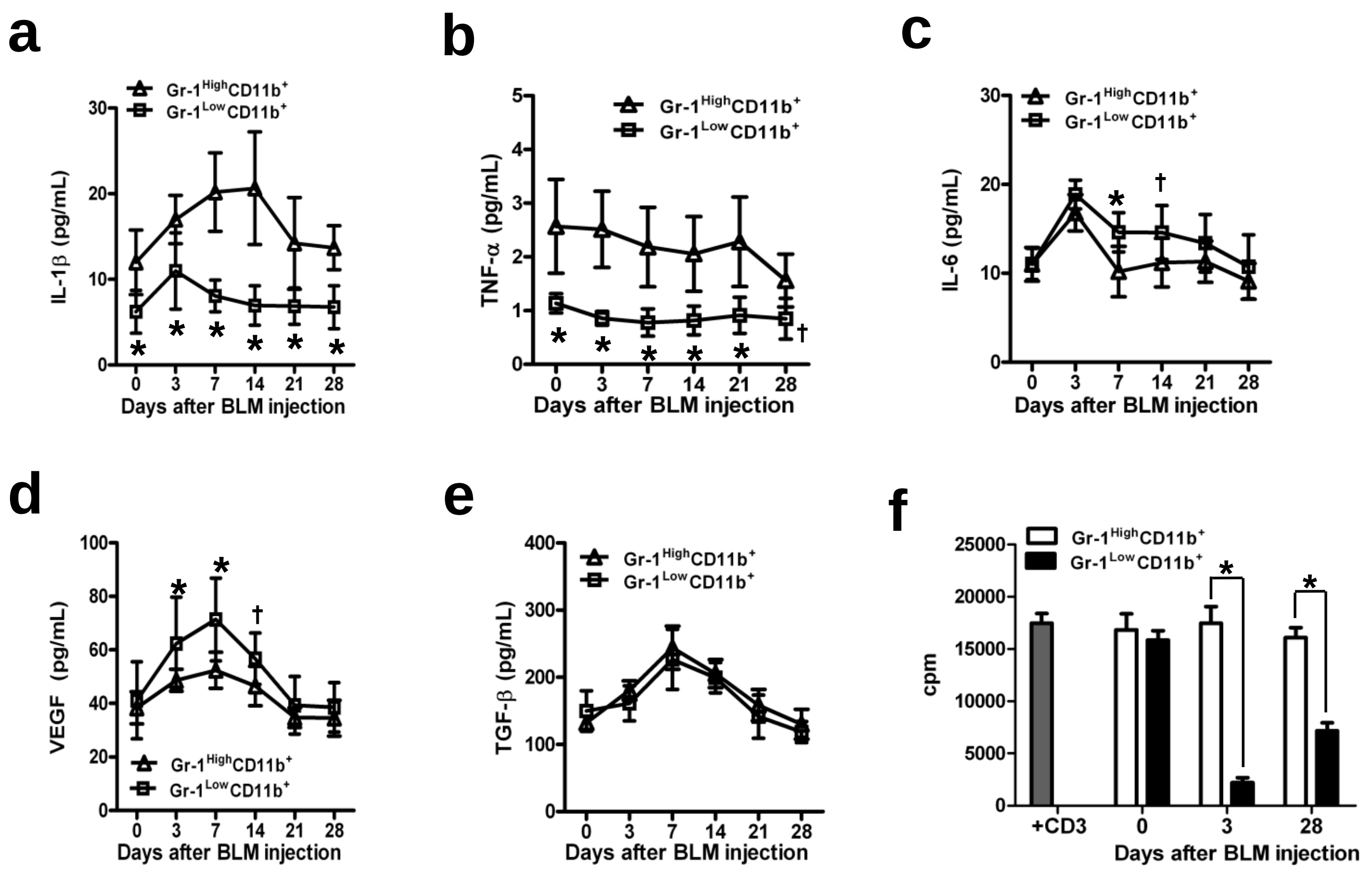

Additional file 6: Figure S6. Pro-inflammatory and Immunosuppressive property of Gr-1^{High}CD11b^{+} and Gr-1^{Low}CD11b^{+} cells. **After BMSC treatment in BLM-treated mice, Gr-1^{High}CD11b^{+} and Gr-1^{Low}CD11b^{+} cells were isolated from lungs of BMSC-treated mice (n=3) and cultured in triplicate (1×10^{5} cells/well). a-e Twenty-four hours later, supernatants were collected and the levels of IL-1β, VEGF, TGF-β, IL-6, and TNF-α were measured by ELISA. **P **< 0.01 and †** P **< 0.05 as compared with "Gr-1^{High}CD11b^{+}" group. f CD3^{+} cells (2×10^{5} cells/well) isolated from spleens of naïve C57BL/6 mice were stimulated with CD3-specific antibodies (1μg/mL) in the presence of Gr-1^{High}CD11b^{+} or Gr-1^{Low}CD11b^{+} cells (1×10^{5} cells/well) purified from lungs of BMSC-treated mice (n=3).** T-cell proliferation was measured in triplicate by ^{3}H-thymidine incorporation. **Data presented**

are representative of two replicated experiments. **P* < 0.01. BLM, bleomycin; BMSC, bone marrow mesenchymal stem cells.
