# Supplementary material for: Bone marrow mesenchymal stem cells tune the differentiation of myeloid-derived suppressor cells in bleomycin-induced lung injury
Source: Stem Cell Res Ther. 2018 Sep 26;9:253. doi: 10.1186/s13287-018-0983-1 (PMC6158827; doi:10.1186/s13287-018-0983-1)
Supplement: Supplementary file 7 — Figure S7. Human BMSC tune the differentiation of mouse MDSC in vitro. (PDF 100 kb) [file 13287_2018_983_MOESM7_ESM.pdf]

Additional file 7: Figure S7

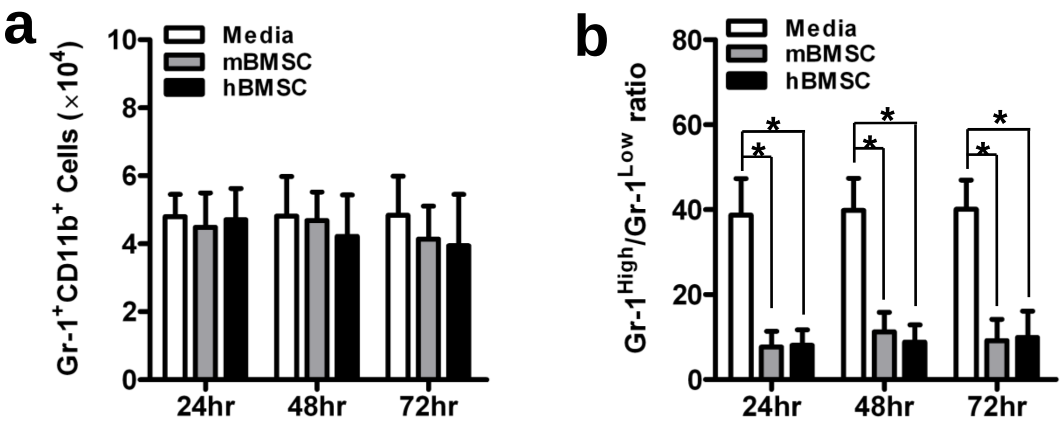

Additional file 7: Figure S7. Human BMSC tune the differentiation of mouse MDSC in vitro. Gr-1^{+}CD11b^{+} cells (5 × 10^{4}/well) isolated from naïve C57BL/6 mice were cultured in RPMI 1640 medium, alone or in the presence of mouse BMSC (mBMSC) or human BMSC (hBMSC) (1 × 10^{4}/well). A total of 24, 48 and 72 hr after coculture, floating cells were collected and numerated. a The absolute number of Gr-1^{+}CD11b^{+} cells and (b) the variations of Gr-1^{High}CD11b^{+}/Gr-1^{Low}CD11b^{+} were calculated. *P < 0.01. BMSC, bone marrow mesenchymal stem cells; mAb, monoclonal antibody; W/O, without.
